# PROMOTING POSITIVE AGING IN COMMUNITY-DWELLING SOUTH ASIAN AMERICANS: AN EXPLORATORY QUALITATIVE STUDY

**DOI:** 10.1093/geroni/igac059.1918

**Published:** 2022-12-20

**Authors:** Mushira Khan, Sheetal Shah, Ajla Basic

**Affiliations:** Mather Institute, Evanston, Illinois, United States; University of California (Davis), Sacramento, California, United States; Mather Institute, Evanston, Illinois, United States

## Abstract

Between 2010 and 2017, South Asians were the fastest-growing major ethnic minority group in the US, growing at a rate of 40% over the previous decade (SAALT, 2019). This exponential growth, along with a rapidly aging US population, implies that a significant proportion of the South Asian American population will be 65 years or older in the coming years; yet research on the lived experiences/needs of older South Asian Americans is limited. To address this gap, this qualitative study explored barriers and facilitators to healthy or positive aging in a sample of community-dwelling South Asian Americans 50 years and older. In-depth, semi-structured interviews were conducted with 32 South Asian American older adults (18 women and 14 men). Thematic analysis of the interview data showed that level of acculturation, proficiency in English, cultural beliefs/practices, awareness about available health and social services, degree of religiosity, and the density of social networks were key determinants of healthy aging. Compared to those who were US-born or had immigrated earlier in life, participants who had immigrated later in life (post-retirement) appeared more financially and/or emotionally dependent on their adult children and expressed ambivalence vis-à-vis future caregiving arrangements and intergenerational co-residence. Nearly all participants shared that helping their adult children with childcare, cooking, or other household chores gave them a sense of purpose and made them feel valued. Study findings suggest that along with culturally appropriate programs and policies to support healthy aging, increased volunteering opportunities may enhance subjective well-being in South Asian American older adults.

